# Retraction: Temperature effects on life history traits of two sympatric branchiopods from an ephemeral wetland

**DOI:** 10.1371/journal.pone.0285260

**Published:** 2023-04-26

**Authors:** The PLOS ONE Editors

After publication of this article [1], concerns were raised about overlap with previous studies [2] and [3], unclear reporting of the methodology, discrepancies in reported data, and availability of underlying data.

In response, the authors engaged in extensive discussions with *PLOS ONE* in which they provided additional methodological information and clarified that some of the data for *E*. *braueriana* (Eb) included in [1] have been previously published. Specifically,

- The values for *E*. *braueriana* life history parameters, EHT, EMT (of hermaphrodite), MLS, and sex ratio, in Table 1 of [1] were previously reported in Table 2 of [2] (cited as reference 10 in article [1]).
- The hatching number of trials (n) for *E*. *braueriana* in Table 1 of [1] were previously reported in Table 1 of [2].
- The data underlying the growth and survival curves for *E*. *braueriana* shown in Figs 1B and 2B, respectively, of [1] have been previously used to generate Figs 2 and 3 of [2]; however, the approach used differed, with the analyses reported in [1] using a different constant, which resulted from curve-walking for numerical analysis by adding “1” to each daily survivor percentage from the dataset. This accomplished the exponential curve plotting for the “0” survivor from the non-hatching days in chambers.

The authors apologize that the reuse of the *E*. *braueriana* data set was not clearly indicated in [1], and further note that the previous data has been combined and juxtaposed with new data in [1] for the purpose of new analysis and interpretation.

The *PLOS ONE* Editors consulted with a member of the Editorial Board, who advised that the reporting of the methodology and datasets used in the originally published article are not sufficiently clear, there were major concerns with the equations, and overall the article did not meet *PLOS ONE*’s publication criteria. The Academic Editor also noted that the regression equations used to make predictions about clutch size were not significant in some cases, and the data used for Part A of Figure A in S1 File were not normally distributed, thereby violating the assumptions of the regression. The *PLOS ONE* Editors also consulted with a statistical reviewer who advised that the model being fitted with both EMT and post-EMT datasets, and the combining of these different sets, is not appropriate.

The *PLOS ONE* Editors note that this raises concerns about the validity and reliability of the conclusions around *R*_*0*_, T_G_ and *r* which are derived from the clutch size equation.

The individual-level data was not provided when article [1] was published. The trial-level data for the aquaculture studies were provided during follow-up discussions but the majority of data for the 20 individuals within each trial are not available.

In light of the above concerns with the analysis methods, which raise concerns over the reliability of the reported results, the *PLOS ONE* Editors retract this article.

The *PLOS ONE* Editors apologize that these issues were not identified prior to publication.

WPH agreed with the retraction and stands by the article’s findings. LSC noted that they accept the retraction decision but stand by the article’s conclusions.
